# Action-outcome delays modulate the temporal expansion of intended outcomes

**DOI:** 10.1038/s41598-024-52287-x

**Published:** 2024-01-29

**Authors:** Rohan R. Donapati, Anuj Shukla, Raju S. Bapi

**Affiliations:** 1 Cognitive Science Lab, Kohli Research Centre On Intelligent Systems, International Institute of Information Technology – Hyderabad, Gachibowli, Hyderabad, 500032 India; 2 Thapar School of Liberal Arts & Sciences, Thapar Institute of Engineering & Technology, Patiala, Punjab 147004 India

**Keywords:** Psychology, Human behaviour

## Abstract

The phenomenon of intentional binding pertains to the perceived connection between a voluntary action and its anticipated result. When an individual intends an outcome, it appears to subjectively extend in time due to a pre-activation of the intended result, particularly evident at shorter action-outcome delays. However, there is a concern that the operationalisation of intention might have led to a mixed interpretation of the outcome expansion attributed to the pre-activation of intention, given the sensitivity of time perception and intentional binding to external cues that could accelerate the realisation of expectations. To investigate the expansion dynamics of an intended outcome, we employed a modified version of the temporal bisection task in two experiments. Experiment 1 considered the action-outcome delay as a within-subject factor, while experiment 2 treated it as a between-subject factor. The results revealed that the temporal expansion of an intended outcome was only evident under the longer action-outcome delay condition. We attribute this observation to working memory demands and attentional allocation due to temporal relevancy and not due to pre-activation. The discrepancy in effects across studies is explained by operationalising different components of the intentional binding effect, guided by the cue integration theory. Moreover, we discussed speculative ideas regarding the involvement of specific intentions based on the proximal intent distal intent (PIDI) theory and whether causality plays a role in temporal binding. Our study contributes to the understanding of how intention influences time perception and sheds light on how various methodological factors, cues, and delays can impact the dynamics of temporal expansion associated with an intended outcome.

## Introduction

The sense of agency is the subjective feeling of being in control of one's actions. To measure the sense of agency, one of the methods employed in literature is the idea of intentional binding, where it is understood that if an action is committed by an agent voluntarily, then the subjective interval between the committed action and its effect is perceived to be temporally shorter than the physical interval at which they are separated at^1–4^. One of the mechanisms behind such subjective attraction is the bi-directional relation between voluntary action and its sensory consequence^5^. A few models have tried to explain the idea of intentional binding^6–8^. One of them is the *sensory recalibration model*^9^, in which the brain recalibrates the temporal interval between a voluntary action and its outcome such that it shifts the outcome towards the action. However, a question arises whether the entire outcome shifts in time towards the action during such recalibration or is just the outcome onset that shifts, leading to an expansion of the outcome.

A study from 2017 by Makwana et al. found that it is indeed the outcome onset that expands, therefore causing an expansion of the intended outcome^10^. In the study, the authors implemented a modified version of the temporal bisection task wherein participants chose which coloured circle they would like to see as an outcome. The outcome selection is followed by an action-outcome delay before the outcome is displayed, where either the intended/selected outcome or the other (unintended) is observed. The participants then had to judge whether the observed outcome was closer to a "SHORT" or "LONG" anchor (300ms and 700ms, respectively), which they had learned before the experiment. The study consisted of three experiments with varying action-outcome delays (250ms, 500ms, 1000ms). The results revealed that the outcome expanded toward the action when the delay was 250ms, and as the delay increased, the effect became less pronounced. To establish a link between intentional binding and the perceived duration of the outcome, experimental studies generally satisfy the following two conditions under which the intentional binding effect is well established^3^: (a) the intentional binding effect tends to decrease as the delay between the action and outcome increases, and (b) the intentional binding effect is present only for intention-based or voluntary actions, not for stimulus-based actions. Their experiments^10^ satisfied these two conditions, grounding the results to intentional binding literature.

However, the task used in the study raises concerns about the operationalisation of intention (free will) due to the way participants selected outcomes representing their intentions. The experimental design included a bar-like cue that indicated how frequently participants had chosen a specific colour during the outcome selection phase. This bar was introduced to prevent participants from repeatedly choosing the same outcome (by pressing a single key all the time) and to encourage them to consider all options equally. According to long-standing philosophical traditions dating from figures like Aristotle to Kant and Hegel, if someone was not "free" when they did something, their actions might not be considered intentional^11^. The purpose of the bar was explained as a loose constraint, serving as a cue to prompt participants to think about the colour they desired to see in each trial. However, such a bar in the participants' visual periphery, guiding their outcome selections, could potentially introduce complications or complexities to the concept of intention itself^12,13^ ("prior conscious thought").

Prioritising information is fundamental to human perception, and it is conceivable that the temporal processing of attended information (in this case, outcome monitoring due to the visual cue) is heightened and accelerated while irrelevant information is suppressed^14–17^. It is plausible that the bar acted as a prior cue, leading participants to enhance their attention toward choosing between the two possible outcomes^18^ instead of focusing on the outcome. This, in turn, might have allowed the intended outcome to reach the threshold of consciousness faster, similar to the *prior entry phenomenon*^19,20^, where attending an event could make it appear earlier than a simultaneous unattended event. Since the processing for the intended outcome, elicited by the peripheral cue, starts earlier, the intentional activity triggered by the attended stimulus could add to the activity already triggered by the cue^21^.

Furthermore, intentional binding, in addition to external cues, has been demonstrated to be intrinsically dynamic in terms of how prior and posterior information are distributed, as proposed by the *cue integration theory*^22–25^. According to this theory, the intentional binding effect is developed through two components^26–28^: a predictive component based on prior information and a postdictive component based on posterior information. Depending on the available information, intentional binding might rely more on one component than the other, resulting in variations in overall binding as well as in the relevant action bindings (perceptual shift of the action towards the outcome) and outcome bindings (perceptual shift of the outcome towards the action). The presence of an external cue during action selection could potentially influence the temporal dynamics of the intentional binding effect^29^.

Therefore, considering the influence of external information on intentional binding and the potential mechanisms of faster processing based on available cues, it is plausible that the bar-like prior cue during the intention selection phase could have impacted the observed outcomes in Makwana et al.’s study^10^, resulting in the expansion of the intended outcome within shorter time frames (250ms and 500ms). Additionally, the authors invoke *pre-activation theory*^30–34^ to explain the expansion of an outcome following the shorter action-outcome delay conditions (250ms and 500ms). Although, based on the arguments presented, removing the bar-like cue that facilitated outcome selection tracking might lead to a difference in how the pre-activation of an intended outcome can affect its temporal expansion.

To address this issue, we implemented a modified version of the temporal bisection task by having a set of six immediately recognisable 2D geometric shapes (Circle, Triangle, Square, Rhombus, Parallelogram, and Pentagon) (see Fig. 6 for the shapes). We used random pairs of these shapes on the intentional selection slide (see Fig. 7 for experiment design). This approach circumvented the confounding factor of the bar-like visual cue used to track the two alternative forced choices. With this modification, participants were now presented with a random pair of objects on each trial, allowing them to independently and freely choose either outcome without being influenced by any external information that might impact their free will.

We conducted our version of the temporal bisection task to assess the effect of intention on the temporal expansion of the outcome, using action-outcome delays of 250ms and 1000ms as a within-subject factor in experiment 1 and a between-subject factor in experiment 2. Additionally, this design allowed us to investigate the pre-activation account reported in previous studies. We hypothesised that if the expansion of an intended outcome resulted from intention being pre-activated rather than the effect of the bar-like cue, we would observe a similar, if not more significant expansion of the intended outcome under the 250ms action-outcome delay condition, but not under the 1000ms action-outcome delay condition, just like in the earlier study^10^.

## Results

### Experiment 1 (within subject design – intermixed action-outcome delays)

**Figure 1:**
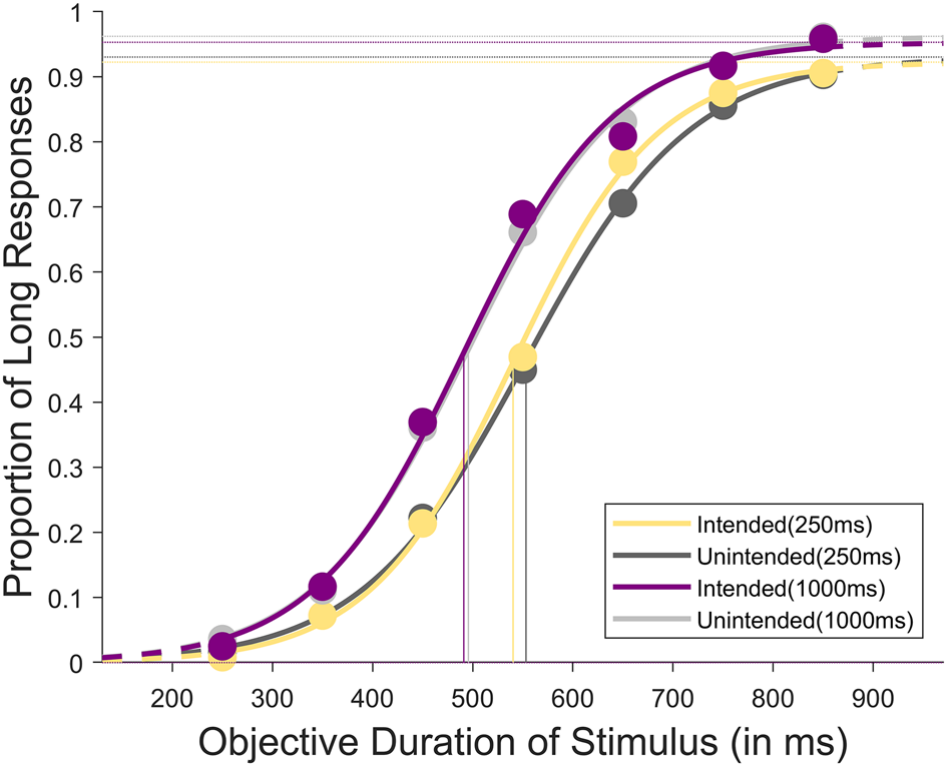
Average psychometric fit for the results of all participants between intended and unintended conditions at an action-outcome delay of 250ms as well as 1000ms from Exp. 1.

In this experiment, we tested whether intention influences the perceived duration of an outcome under different action-outcome delays of 250ms and 1000ms as a within-subject factor. Under the temporal bisection task, participants underwent initial training using two anchor durations, labelled as "SHORT" (250ms) and "LONG" (850ms). Subsequently, during the testing phase, participants engaged in object selection, followed by an action-outcome delay, and finally, the presentation of the outcome (see Fig. 7 for the experimental design). The participants were tested with seven objective durations, ranging from 250 to 850ms in 100ms increments, which served as comparison stimuli. Their task was to judge whether the observed target stimuli were closer in duration to the "SHORT" or "LONG" anchor. The outcome being intended or unintended, and the action-outcome delay was set at a chance level across all trials. The collected data were then organised based on each participant's two outcome conditions (intended and unintended) and the two action-outcome delays (250ms and 1000ms). To analyse the data, we fitted each participant's responses to a logistic psychometric function (see Fig. 1 for the average fit of all participants). We estimated the bisection points (BPs) and the difference limen (DL) from this function (see supplementary information file 1 for the relevant BPs and DLs). The bisection point represents the duration at which the probability of perceiving the observed outcome as "long" is 50%. On the other hand, the difference limen is a measure of precision, also known as the "just noticeable difference", corresponding to half the difference between the values at 75% and 25% probabilities of responding "long". We considered an outcome expansion when the intended outcome showed a leftward shift in the bisection point compared to the unintended outcome.

**Figure 2:**
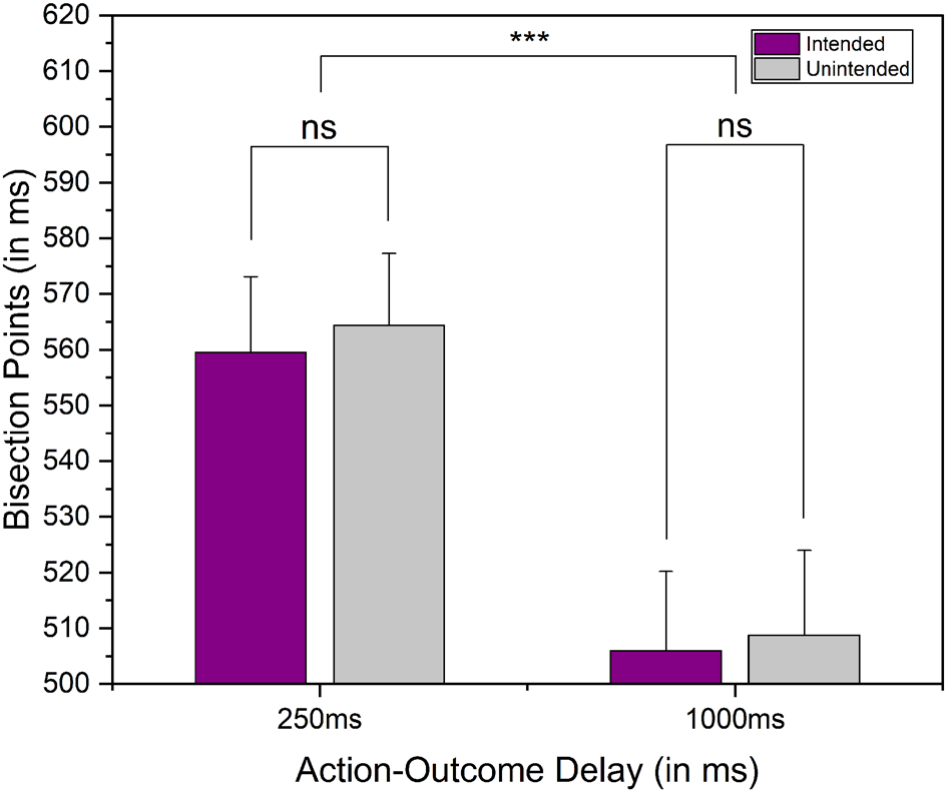
Results of Experiment 1. Comparison of average BPs (Bisection Point) under the intended and unintended conditions across the two action-outcome delays as a within-subject factor. The error bar represents the standard error of the mean, ***indicates p < 0.001, and ns indicates p > 0.05.

A 2 (Outcome: Intended and unintended) × 2 (Action-Outcome Delay: 250ms and 1000ms) within-subject repeated measures ANOVA on BP values showed no significant effect of intention [F(1,22) = 0.608, p = 0.444, ${\upeta }_{{\text{p}}}^{2}$= 0.027] as well as intention x delay interaction [F(1,22) = 0.036, p = 0.852, ${\upeta }_{{\text{p}}}^{2}$= 0.002], indicating that participants did not perceive the intended event as longer than the unintended event (see Fig. 2 for average BPs). However, the delay seemed to have a significant effect [F(1,22) = 32.87, p < 0.001, ${\upeta }_{{\text{p}}}^{2}$= 0.599]. A similar ANOVA on the DLs showed no significant effect of intention [F(1,22) = 0.825, p = 0.373, ${\upeta }_{{\text{p}}}^{2}$ = 0.036], delay [F(1,44) = 2.398, p = 0.136, ${\upeta }_{{\text{p}}}^{2}$ = 0.098], and intention x delay interaction [F(1,44) = 0.263, p = 0.613, ${\upeta }_{{\text{p}}}^{2}$ = 0.012].

We also subjected the observed bisection points (BPs) to a Bayesian paired sample *t*-test to assess the strength of the null effect of intention in both the action-outcome delay conditions. For the Bayesian paired sample *t*-test, we utilised a standard Cauchy prior with a scale parameter of 0.707 for a one-tailed alternative hypothesis (H1: Intended BP < Unintended BP). The calculated Bayes factor indicated that the data were 2.46 times more likely to align with the null hypothesis than the alternative hypothesis under the 250ms action-outcome delay condition. Similarly, under the 1000ms action-outcome delay condition, the data were 3.42 times more likely to support the null hypothesis than the alternative hypothesis. This provides moderate evidence for the absence of a significant effect of intention in both delay conditions.

The findings indicate that participants did not perceive any difference in the outcome duration between the intended and unintended trials across the two delay conditions. However, it appears that the action-outcome delay itself has an influence. Specifically, when the delay was set at 1000ms, participants tended to overestimate the outcomes consistently, regardless of whether they were intended or unintended (see Fig. 2 for average BPs).

The predictability and timing of events play a role in intentional binding^7,35–38^, and the lack of significant intentional effects in Experiment 1 could be attributed to the erratic timing variations between actions and outcomes. Nevertheless, there have been experimental instances where intentional binding was observed even in situations with unpredictable action-outcome delays^39–42^. However, considering the context of the study, where we are trying to probe the pre-activation account, it is reasonable to explore this phenomenon under consistent action-outcome delays in our research.

### Experiment 2 (between subject design)

**Figure 3:**
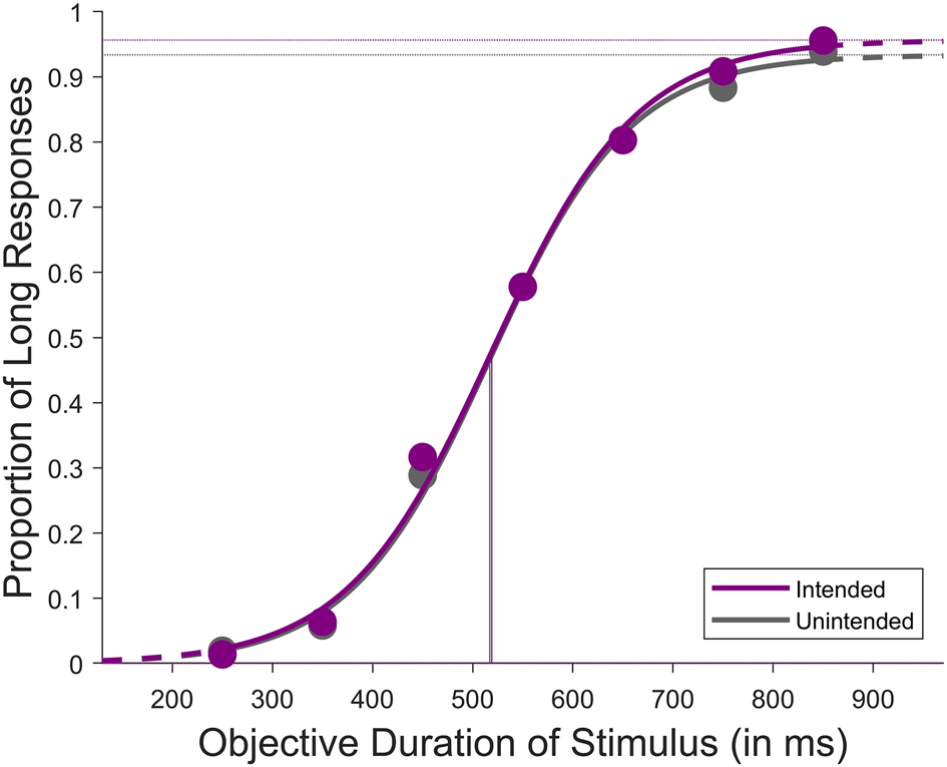
Average psychometric fit for the results of all participants between intended and unintended conditions at an action-outcome delay condition of 250ms from Exp. 2.

**Figure 4:**
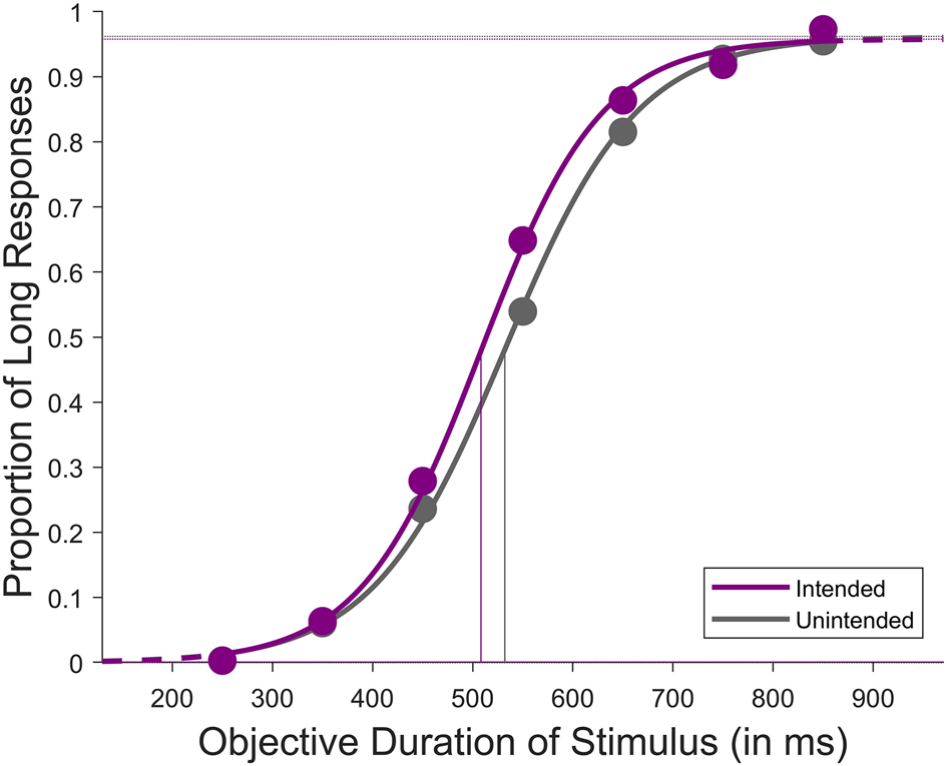
Average psychometric fit for the results of all participants between intended and unintended conditions at an action-outcome delay condition of 1000ms from Exp. 2.

Like experiment 1, the participants in this experiment also performed the temporal bisection task (see Fig. 7 for the experiment design). However, in contrast, we implemented the action-outcome delay as a between-subject factor for this experiment. This allowed us to examine the effect of action-outcome delay on intention independently. The participants were divided into two groups, and each group was tested with one of the two action-outcome delays (250ms or 1000ms). The collected data were first organised based on the two outcome conditions (intended and unintended). Each participant’s data was fitted to a logistic psychometric function (see Figs. 3 and 4 for the average fits of all participants at the two delay conditions), and the BPs were estimated along with the DLs (see supplementary information file 2 for relevant BPs and DLs).

**Figure 5:**
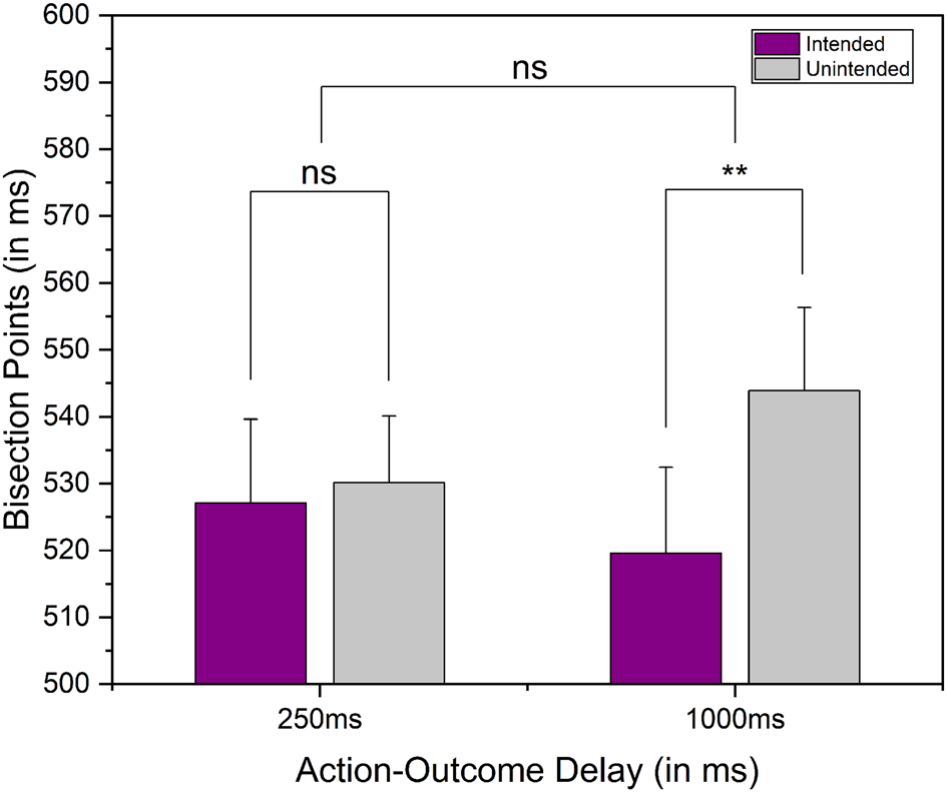
Results of Experiment 2. Comparison of average BPs (bisection points) under the intended and unintended conditions across the two action-outcome delays as a between-subject factor. The error bar represents the standard error of the mean, **Indicates p < 0.01, and ns indicates p > 0.05.

Repeated measures 2 (outcome: intended and unintended) × 2 (action-outcome delay: 250ms and 1000ms) mixed ANOVA with the action-outcome delay being a between-subject factor and the outcome being a within-subject factor, showed a significant effect of intention [F(1,44) = 8.096, p = 0.007, ${\upeta }_{{\text{p}}}^{2}$ = 0.155] and a significant effect of intention × delay interaction [F(1,44) = 4.906, p = 0.032, ${\upeta }_{{\text{p}}}^{2}$ = 0.100]. However, there was no significant effect of delay [F(1,44) = 0.037, p = 0.849, ${\upeta }_{{\text{p}}}^{2}$ = 8.374e^–4^]. Tukey corrected post hoc tests show that there was an effect of intention under the 1000ms action-outcome delay condition [p = 0.006] only (519.578 ± 60.161ms under intended and 543.862 ± 58.563ms under unintended) and not in the 250ms action-outcome delay (527.093 ± 61.42ms under intended and 530.119 ± 48.875ms under unintended) (see Fig. 5 for average BPs). A similar mixed ANOVA on the DLs showed no significant effect of intention [F(1,44) = 2.196, p = 0.145, ${\upeta }_{{\text{p}}}^{2}$ = 0.048], delay [F(1,44) = 1.567, p = 0.217, ${\upeta }_{{\text{p}}}^{2}$ = 0.034], and intention x delay interaction [F(1,44) = 1.59, p = 0.214, ${\upeta }_{{\text{p}}}^{2}$ = 0.035]. To evaluate the strength of the null effect of intention under the 250ms delay condition, we performed a Bayesian paired sample *t*-test on the observed BPs. For the Bayesian paired sample *t*-test, we utilised a standard Cauchy prior with a scale parameter of 0.707 for a one-tailed alternative hypothesis (H1: Intended BP < Unintended BP). The calculated Bayes factor indicated that the data were 3.14 times more in favour of the null hypothesis, providing moderate evidence for the absence of a significant effect of intention.

## Discussion

There is a growing body of research exploring the influence of intention on perception^43–46^. One such study used a unique approach to the temporal bisection task to demonstrate temporal expansion when individuals had a specific intention for an outcome^10^. However, questions can be raised about the robustness of these results concerning the influence of prior information available to participants and how it affects the operationalisation of intention. To address this, our current study aimed to investigate the role of intention on temporal expansion while modifying the adaptation previously introduced in the temporal bisection task. We accomplished this by eliminating any confounding factors related to the voluntary action selection process. In our experiment, participants were asked to choose a 2D, filled geometrical object as part of the outcome selection and then monitored the duration of either the object they chose or the other to make a judgment about its duration compared to previously memorised temporal anchors (250ms as SHORT or 850ms as LONG). To examine the effect more closely, we presented the objects after either a short delay of 250ms or a longer delay of 1000ms. Experiment 1 employed the action-outcome delay as a within-subject design, while Experiment 2 employed a between-subject design to explore these phenomena further.

The results from the initial experiment, where the action-outcome delay was a within-subject factor, did not reveal any intention-induced expansion of the outcome in either of the action-outcome delay conditions. Nonetheless, it is worth noting that there was a general tendency to overestimate the outcome under the 1000ms delay condition, regardless of whether the outcome was intended or not. This indicates that participants experienced an expansion effect for the outcome in the 1000ms delay condition, irrespective of their intention (see Fig. 2 for average BPs). On the other hand, the results from the second experiment revealed a different outcome. Here, an effect of intention on the temporal expansion of the outcome was observed. However, this effect was specifically seen in the context of the 1000ms action-outcome delay condition (See Fig. 5 for average BPs).

Makwana et al.’s earlier study^10^ demonstrated an intention-induced expansion effect for shorter delays (250ms and 500ms) but not for longer delays (1000ms). They attributed these findings to a pre-activation account of intended outcomes, which suggests that self-generated expectations cause a form of pre-activation^30–34^ of the representation of an intended outcome. This pre-activation leads to a faster accumulation of its awareness threshold, resulting in these outcomes being experienced earlier. According to this account, pre-activations of self-generated expectations are more substantial than cue-induced expectations^47–49^. However, the results of the current study contradict this explanation. Suppose the expansion of an intended outcome was solely due to the pre-activation of intention. In that case, an expansion effect should have been observed even for the 250ms action-outcome delay in either of the experiments. However, that was not the case; the expansion effect was evident only after a substantial delay (1000ms). This suggests that the expansion observed in this study is not attributable to a pre-activation account but rather to other factors.

A shorter delay between stimuli can result in rapid succession, potentially hindering the encoding of intentions. The immediate presentation of stimuli may challenge the cognitive processes involved in forming stable intentional representations, making it difficult to retrieve intentions effectively. Shorter delays could also impede the development of the intentional representation and impose an increased cognitive load and demands on working memory, further disrupting the stable representation of intentions and compromising intention recognition. Conversely, a longer delay gives individuals more time for intentional processing and representation^50^. Intentional encoding may occur more accurately during longer delays as individuals have sufficient time to form and maintain intentions. This temporal spacing of stimuli may facilitate intentional representation, leading to improved intention recognition. Hence, the dependence on the delay can explain why we only observe an effect of intention after a longer action-outcome delay.

In addition to the effect of intention after a longer delay, there was an expansion of the outcome when it aligned with participants' intentions. Studies on time perception suggest that when participants concurrently perform a non-temporal task (such as recognising intention) during prospective timing, they tend to overestimate duration judgments when they prioritise timing as the main task^51^. This is consistent with established models of prospective timing^52^, where focusing on time can lead to an overestimation of prospective duration judgments. However, the choice of what to focus on during prospective duration judgments can be influenced by the cognitive system's needs in relation to the current task's goal^53^.

In our case, when the participants choose an outcome, it was in line with their goal of encountering the chosen outcome later. As a result, the relevance they assigned to a specific outcome selection determined the depth of information processing and the amount of resources allocated to its prospective judgment^54^. Hence, it is possible that when participants experienced an intended outcome, temporal relevance played a role in directing attentional resources toward its judgment, making the event feel longer.

According to the temporal relevance model of prospective time judgments^55^, the level of temporal relevance influences the amount of attention allocated to a particular duration judgment. When temporal relevance surpasses a certain threshold, attentional resources are directed toward the duration judgment, while minimal allocation occurs when temporal relevance is not significant (below the threshold). This attentional allocation based on temporal relevance enhances temporal information processing. Since participants in our study were actively anticipating an intentional outcome, it is plausible that their judgments were overestimated when they experienced it. Therefore, the observed expansion effect resulting from intention in the second experiment may be attributed to the retrieval of intention from memory and the engagement of attention during the task due to its temporal relevance.

Apart from our primary exploration of the effect of intention on the temporal expansion of an outcome, we made two intriguing observations in the first experiment. (a) the significant effect of delay on the BPs, and (b) the non-significant effect of intention (see Fig. 2 for average BPs).

Recent research on the intentional binding effect has raised concerns about a potential confounding factor in its measurement. Some studies have criticised the binding effect, suggesting it might be a by-product of the experimental task design^56–59^. Binding effects are compared based on the presence or absence of overt actions, which can lead to a confounded attribution of the binding effect solely to intention. This is especially true as there have been experimental studies where the mere execution or even the resemblance of an action could cause a temporal binding effect similar in magnitude to what is observed in traditional intentional binding studies, even without any semblance of intention present^60–62^.

Studies that have observed a binding effect regardless of intention tend to explain the effect arising from the perception of a causal association between the action and the outcome^62–64^. The significant effect of delay and the absence of intentional effects observed in experiment 1 could also be attributed to such a causal association between the action and the outcome, and the lack of significance in the difference limens (DLs) supports this interpretation. According to a recent study by Fereday et al.^65^, the temporal acuities (precision or DL) should remain consistent across causal conditions when comparing two causal conditions. The results from our first experiment align with the suggestion made by Fereday et al. This could indicate that the observed results in our experiment 1 might be attributed to causal multisensory integration rather than solely to intentional factors. Even though we had attributed the expansion of the outcome in the second experiment as due to intention, this does not negate the role of causality in this context.

It is plausible that the results we observe in experiment 2 can be interpreted as temporal binding relying on causality and intentions serving as a mechanism to establish causal congruency. Experimental evidence supports this notion as well, as it indicates that temporal binding in the context of causal events produces a more substantial binding effect when participants believe they were the ones who performed the action^66,67^. Consequently, intentional binding may be a part of a broader phenomenon of causal multisensory integration, where the cause of an event happens to be intentional. Nevertheless, additional experimentation is necessary to further probe the causal role in intentional binding.

The non-significant effect of intention in experiment 1 may also be attributed to the nature of the task. The continuous recalibration of outcome anticipation^9^ or learning of contingencies^37,68^ might have overshadowed intentional binding due to the unpredictable nature of action-outcome delays. While some studies have demonstrated significant binding even with unpredictable action-outcome delays^39–42^, meta-analytic reviews suggest that temporal predictability plays a crucial role in intentional binding^7,69^, particularly in the context of the Libet Clock method. However, considering that the Libet Clock method engages different processes than inferential methods^70^, we need to be careful while considering this.

Previous studies that used the interval estimation task also indicated some influence of temporal predictability on temporal estimates. For instance, research conducted by Imaizumi et al.^40^ and Humphreys et al.^71^ differed in how action-outcome delays were manipulated. In the former, delays varied within a block of trials, and though they found a significant binding effect, further analysis showed that the binding weakened as the action-outcome delay increased. In contrast, the latter study demonstrated a significant interaction between action-outcome delay and the amount of observed binding, with binding increasing as the action-outcome delay increased. Moreover, a follow-up experiment by Imaizumi et al. using visual outcomes instead of auditory ones resulted in the disappearance of the binding effect. Another study by Nolden et al., which measured intentional binding using the method of constant stimuli, found more significant Weber fractions when participants experienced different action-outcome delays within the same block of trials, suggesting reduced performance under changing action-outcome delays^72^.

While the evidence from these studies is not entirely conclusive, the presented data leads us to believe that the temporal predictability of outcome onsets may have influenced the results observed in our first experiment. This aligns with the findings of the meta-analyses mentioned above, which emphasised that the temporal context, specific characteristics of the action and outcome context, and the type of outcome modality (visual, haptic, auditory) can induce different modulation of binding. Alternatively, it could be possible that another factor might be interfering with the intentional representation. Selecting a response and recognising its congruence with the chosen object involves a conscious and controlled process. Consequently, this process could be influenced by cognitive factors such as attention, task requirements, or a shortage of cognitive resources^73^. It is possible that the unpredictability of the action-outcome delay heightened attentional demands, leading participants to focus more on calibrating for the outcome's timing rather than recognising the intentional connection, akin to a form of choice blindness^74,75^.

Our experiment showed an interesting outcome related to the impact of intention following longer delays. This aligns with the binding dynamics commonly observed in inferential paradigms used to measure the intentional binding effect^65,71,72,76–78^. These paradigms typically show significant binding effects after longer action-outcome delays^79^. In our study, the absence of an external cue may have caused participants to focus more on the outcome and its connection to the committed action or intention rather than on the action selection process. Conversely, when external cues are present, participants may become more action-specific in their focus, leading to results that align with sensory-based paradigms like the Libet clock method^1,3,38^, where binding effects tend to be short-lived and operate within a strict time window^80^.

The differential impact of delay on intentional binding is commonly linked to the methodology employed as well as what components of the intentional binding effect are engaged^26–28,70^. Sensory paradigms show significant binding effects under shorter action-outcome delays, while inferential paradigms reveal such effects under longer delays^40,79^. However, studies like Ruess et al.'s experiment indicate that the same methodology can demonstrate varying sensitivities of the binding effect depending on the action-outcome delay^38,81^ and other external factors^24^. The differential sensitivity to components of the intentional binding effect may be explained by the Bayesian cue integration theory^22–25^. According to this theory, the observed temporal binding between an action and its outcome results from an optimal Bayesian integration of information^82,83^. Based on the limited evidence available, we can speculate that Makwana et al.’s study engaged the processes relevant to the Libet clock method, and in our study, inferential processes were given priority.

While our experimental design did not directly assess this aspect, recent research suggests that intentional operationalisation through experimental design could influence the binding effect^84,85^. Philosophical literature on the phenomenology of intention^86^ discusses a distinction between proximal and distal intentions. Proximal intent focuses on the mechanics of performing an action, while distal intention involves the broader purpose beyond the action's execution.

The PIDI framework (proximal intent distal intent) suggests that one form of intent can be more prominent than the other^87^, similar to the dual component theory underlying the intentional binding effect. Proximal intentions align with the Libet method for measuring the intentional binding effect, whereas distal intentions align with inferential methods. The observed expansion of outcomes in our second experiment, specifically at the 1000ms action-outcome delay, may be attributed to a stronger emphasis on distal intention since distal intentions require the establishment of intention in working memory^88^, and it is understood that proximal intentions characterise the Libet method for measuring the intentional binding effect^89,90^. The variation in delay effects across the studies (Makwana et al.’s and ours) could be linked to the specific operationalisation of intention and the engagement of components underlying the intentional binding effect. Sensory-based proximal intention may cause outcome expansion at shorter delays but decays for longer delays. On the other hand, inference-based distal intention causes outcome expansion at longer delays.

In conclusion, we conducted two experiments to examine the pre-activation account of intention on the temporal expansion of an intended outcome. We found a significant effect of outcome expansion due to intention. However, this effect was observed only in the 1000ms action-outcome delay condition and when the outcome onset was predictable. We attribute this observation to working memory demands and attentional allocation due to temporal relevancy. The differential effect of action-outcome delays across the two studies can be explained by considering the operationalisation of different components of the intentional binding effect through the cue integration theory, and we speculate there might also be variations in the operationalisation of intentions. Moreover, intentional binding could be a subset of a more extensive set of factors (including causality) contributing to temporal binding. With the inclusion of causality, intentional binding, which has traditionally been described as a manifestation of a general linkage through time, could now be seen as a potential bi-directional ambiguity reduction mechanism.

Moreover, addressing the critique of free will^91^, our study focused more on the outcome than on action selection, allowing us to measure the expansion of a genuinely endogenous, internally generated intention, which involves more descriptive and abstract levels of intention beyond mere motor processes. It is crucial to be mindful of relevant perceptual cues, task methodology, and action-outcome delays, as they can influence the dynamics of intentional binding development.

Future research could further scrutinise the operationalisation of intention and investigate the reasons behind this subjective expansion using well-established time perception models like the clock model^92–94^. Exploring which component (pacemaker or switch/gate) contributes to the temporal expansion of an intended outcome would also be valuable. Additionally, understanding the interaction between causality and intention could provide deeper insights into the mechanisms of intentional binding.

## Methods

### Participants

A total of 23 participants in experiment 1 (mean age: 24, 5 female) and 46 participants in experiment 2 (mean age: 22, 15 female) (24 participants in the 250ms delay group and 22 participants in the 1000ms delay group) were recruited from the International Institute of Information Technology, Hyderabad, India. Although we did not perform an apriori sample size estimation, we referenced a similar study^10^ that utilised 14 participants in a between-subject design to investigate the influence of intention on the temporal processing of outcomes. Considering our study's similarity and distinctive within-subject design, we recruited 23 participants for experiment 1 and 46 participants for experiment 2 (between-subject design) in our study. All participants were healthy and naïve as to the purpose of the study. They had normal or corrected-to-normal vision, and the Institute Review Board (IRB), International Institute of Information Technology, Hyderabad, India, approved the study. All the experimental procedures and methods were performed per the relevant guidelines and regulations. Informed consent forms were obtained from all the participants, and remuneration was paid for their participation.

### Stimuli and apparatus

**Figure 6:**
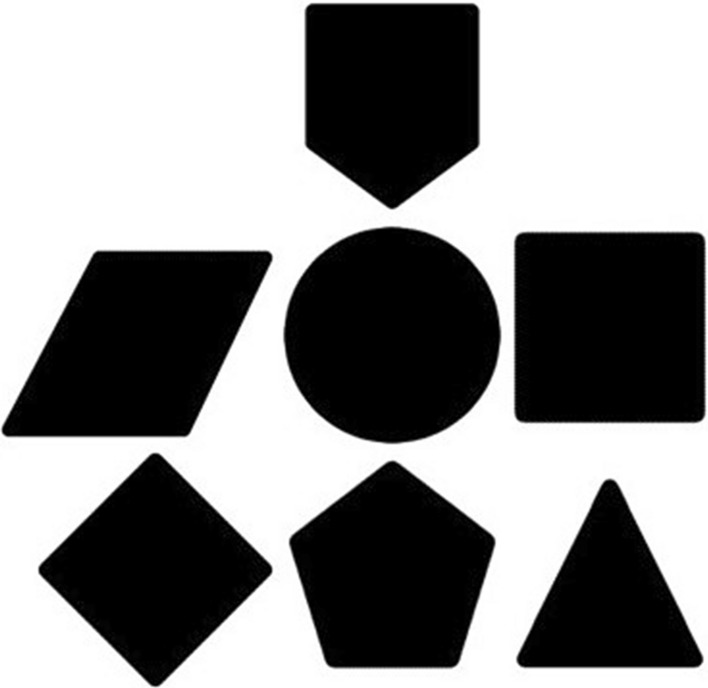
Set of six solid 2D geometric shapes used as selectable outcomes in the testing phase of the experiment (Parallelogram, Circle, Square, Rhombus, Pentagon, Triangle) along with a badge-shaped object for training and feedback phases of the experiment.

All experiments were designed using Psychtoolbox (Ver. 2.0.18) on MATLAB(R2021b) and run on a CRT monitor (1024 × 768 resolution) at a refresh rate of 100Hz. Participants sat 60cm from the monitor screen in a dimly lit experimental room. Stimuli consisted of six solid black 2D, each sized 150 × 150 pixels, with a visual angle of 3.82 degrees against a white background. During both experiments' training and feedback phases, an additional badge-shaped 2D object was used in addition to the main six objects (see Fig. 6). To analyse the data, the psychometric curve was fitted using the Psignifit toolbox (Ver. 2.5.6) in MATLAB. All other analyses were performed using JASP (Ver. 0.16).

### Procedure

**Figure 7:**
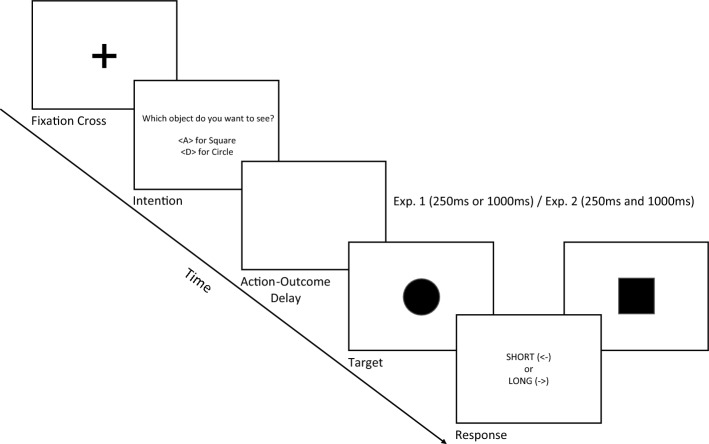
Experimental design employed in both experiments. Before each trial, the participants select the object they want to see by pressing the pre-assigned keys. Outcome selection was followed by either a 250ms or 1000ms action-outcome delay in experiment 1 (delay as a within-subject factor) and experiment 2 (delay as a between-subject factor). The target stimulus would randomly be either the intended or unintended selection. This target was flashed for a random objective duration between 250 to 850ms in steps of 100ms, after which the participants reported whether the target stimulus was closer to the LONG (850ms) anchor or SHORT (250ms) anchor by pressing the relevant arrow keys.

We employed a modified version of the temporal bisection task for all experiments (see Fig. 7). The trial structure consisted of three phases: the training phase, the feedback phase, and the testing phase. In the training phase, participants encountered a flashing badge-shaped 2D object ten times for a SHORT duration of 250ms and ten times for a LONG duration of 850ms. They were instructed not to use counting strategies but to develop a mental representation of the learned temporal anchors. During the feedback phase, participants were required to identify the SHORT or LONG anchor durations they learned in the training phase with an accuracy above 95%.

In each self-paced trial of the testing phase, participants were presented with a fixation cross, followed by two objects labelled "Object 1" and "Object 2”. They had to choose between the two objects by pressing a designated key corresponding to the word representing the pair of objects in that particular trial. Words were used instead of shapes to allow quick assessment and activation of the representation of the shape before making a selection^95^. To ensure participants recognised the 2D shapes, they were asked to match the shapes on the screen with their corresponding names before the experiments. Participants were explicitly instructed to base their key press on the object represented by the key, not the key itself.

Once the participant selects an object, one of the two action-outcome delays was followed by either the intended selection or the other for one of seven objective durations ranging from 250 to 850ms in steps of 100ms increments. The probability of getting the intended outcome was set at chance (50%) to prevent accurate prediction of the target object, thereby ensuring that any observed effects were attributed to intention rather than prediction. Participants reported the duration of the object as closer to the SHORT (250ms) or LONG (850ms) anchor they learned in the training and feedback phases of the experiment by pressing the corresponding key. Four breaks were incorporated between the trials, corresponding to each experiment's total number of trials. Participants' intention response, i.e. what object they wanted to see, and their duration judgment response (SHORT/LONG) were recorded.

In experiment 1, the target stimulus appeared either after a 250ms delay or a 1000ms delay, interspersed in a within-subjects design, resulting in 420 trials. There were 210 trials under each delay condition (250ms and 1000ms), half of which were intended selections and the rest unintended. Each objective duration (250ms to 850ms) had 15 trials. The six objects were paired based on a 6 × 6 arrangement, with repetitions removed, resulting in 30 combinations repeated seven times, leading to 210 object pairs per action-outcome delay. All factors were completely randomised.

In experiment 2, the action-outcome delays of 250ms and 1000ms were considered a between-subjects factor, meaning participants belonged to either the 250ms or 1000ms delay group. Each participant underwent 210 trials, of which 105 were intended, with 15 trials per objective duration. The 210 object pairings were randomised within each group.

### Supplementary Information

Supplementary Information 1.

Supplementary Information 2.

## Data Availability

All data generated or analysed during this study are included in this published article [and its supplementary information files]. Specifically, supplementary information file 1 contains data (BPs and DLs) from experiment 1 and supplementary information file 2 contains data (BPs and DLs) from experiment 2.
